# Unraveling the Complexity of Cardiac Distress: A Study of Prevalence and Severity

**DOI:** 10.3389/fpsyt.2022.808904

**Published:** 2022-03-31

**Authors:** Alun C. Jackson, Michelle C. Rogerson, John Amerena, Julian Smith, Valerie Hoover, Marlies E. Alvarenga, Rosemary O. Higgins, Michael R. Le Grande, Chantal F. Ski, David R. Thompson, Barbara M. Murphy

**Affiliations:** ^1^Australian Centre for Heart Health, Melbourne, VIC, Australia; ^2^Center on Behavioral Health, Faculty of Health, Deakin University, Geelong, VIC, Australia; ^3^Behavioral Health, University of Hong Kong, Pokfulam, Hong Kong SAR, China; ^4^Barwon Health, Geelong, VIC, Australia; ^5^Deakin School of Medicine, University Hospital Geelong, Geelong, VIC, Australia; ^6^Department of Cardiothoracic Surgery, Monash Health, Melbourne, VIC, Australia; ^7^Department of Surgery, School of Clinical Sciences, Monash Health, Melbourne, VIC, Australia; ^8^Psychiatry and Behavioral Sciences, Stanford Women's Heart Health Clinic, Stanford, CA, United States; ^9^School of Health and Life Sciences, Federation University Australia, Berwick, VIC, Australia; ^10^Victorian Heart Institute, Melbourne, VIC, Australia; ^11^Monash Health & Department of Medicine, Monash University, Melbourne, VIC, Australia; ^12^Department of Psychology, Deakin University, Geelong, VIC, Australia; ^13^Department of Physiotherapy, University of Melbourne, Melbourne, VIC, Australia; ^14^Centre for Behaviour Change, Melbourne School of Psychological Sciences, University of Melbourne, Melbourne, VIC, Australia; ^15^Integrated Care Academy, University of Suffolk, Ipswich, United Kingdom; ^16^Department of Psychiatry, University of Melbourne, Melbourne, VIC, Australia; ^17^School of Nursing and Midwifery, Queen's University Belfast, Belfast, United Kingdom; ^18^Department of Psychology, University of Melbourne, Melbourne, VIC, Australia

**Keywords:** psychocardiology, cardiac distress, anxiety, stress, depression, fear of progression, secondary prevention

## Abstract

**Introduction:**

While much research attention has been paid to anxiety and depression in people who have had a recent cardiac event, relatively little has focused on the broader concept of cardiac distress. Cardiac distress is a multidimensional construct that incorporates but extends beyond common mood disorders such as anxiety and depression. In the present study we assessed the prevalence, severity and predictors of a broad range of physical, affective, cognitive, behavioral and social symptoms of cardiac distress. This is the first study to investigate cardiac distress in this comprehensive way.

**Method:**

A sample of 194 patients was recruited from two hospitals in Australia. Eligible participants were those who had recently been hospitalized for an acute cardiac event. Data were collected at patients' outpatient clinic appointment ~8 weeks after their hospital discharge. Using a questionnaire developed through a protocol-driven 3-step process, participants reported on whether they had experienced each of 74 issues and concerns in the past 4 weeks, and the associated level of distress. They also provided sociodemographic and medical information. Regression analyses were used to identify risk factors for elevated distress.

**Results:**

Across the 74 issues and concerns, prevalence ratings ranged from a high of 66% to a low of 6%. The most commonly endorsed items were within the domains of dealing with symptoms, fear of the future, negative affect, and social isolation. Common experiences were “being physically restricted” (66%), “lacking energy” (60%), “being short of breath” (60%), “thinking I will never be the same again” (57%), and “not sleeping well” (51%). While less prevalent, “not having access to the health care I need,” “being concerned about my capacity for sexual activity,” and “being unsupported by family and friends” were reported as highly distressing (74, 64, and 62%) for those experiencing these issues. Having a mental health history and current financial strain were key risk factors for elevated distress.

**Conclusion and Implications:**

Specific experiences of distress appear to be highly prevalent in people who have had a recent cardiac event. Understanding these specific fears, worries and stressors has important implications for the identification and management of post-event mental health and, in turn, for supporting patients in their post-event cardiac recovery.

## Introduction

The psychosocial impacts of an acute cardiac event have gained increasing recognition in the past two decades. It is now well-accepted that both anxiety and depression are common after heart attack and heart surgery (1–3). In a recent Australian study involving over 900 patients admitted to hospital after acute myocardial infarction (AMI) or to undergo coronary artery bypass graft surgery (CABGS), over 40% had elevated anxiety and over 20% had symptoms of depression in the period shortly after hospital discharge, while 31% had anxiety or depression at 6–12 months post-event (3). These rates are up to four times higher than in the general population.

There is also growing evidence that acute cardiac events are often experienced as traumatic, thereby predisposing survivors to experiencing post-traumatic stress symptoms (PTSS) or disorder (PTSD). The traumatic components of a cardiac event include its abruptness, the risk of death, and a sense of helplessness and loss of control during and after the event (4). It has been suggested that around 12% of patients experience acute coronary syndrome (ACS)-induced PTSD (5).

But is there more to a cardiac event than just anxiety, depression and PTSD? Qualitative studies involving cardiac event survivors and clinicians who support them provide greater insight into the breadth of experiences, concerns and worries expressed during convalescence after an acute cardiac event and therefore provide a nuanced understanding of cardiac distress. One qualitative study of survivors concluded that they experience “being forced into a demanding life-shaking journey” (6), while another study of clinicians described it as “a lonely journey, an existential crisis” (7). It appears that the acute cardiac event can trigger the beginning of a completely new life chapter, involving new and difficult emotions, changes in self-concept and identity, and other unexpected challenges, fears and concerns.

Reported experiences gleaned from qualitative studies include a range of emotions, such as feelings of uncertainty (8–10), vulnerability (6), loneliness and fear of being alone (7), hopelessness and helplessness (6, 7), anger and resentment (7), sadness, grief and loss (7, 8). Specific losses include loss of independence (7, 11), loss of health and physical strength (8, 11), and loss of control (7). Similarly, various changes in self-identify and self-concept have been reported (12), as have worries about getting back to one's previous sense of self (13). These are often tied to changes in roles and role function (8, 12), including loss of the ability to provide (11) or be the “breadwinner” or “homemaker” (7). Impacts on intimate relationships are also a concern for some survivors (13), including concerns about resuming sexual activity due to fear of causing another heart attack (7, 14). Challenges navigating the health system are also evident, including difficulties in obtaining information and advice (13), and concerns about having to rely on help from health professionals (9).

The cardiac event also triggers difficulties in coping with change (9), including difficulties adjusting to limitations in everyday life (6) and living with pain (7), resistance to being on medications (9), hypervigilance regarding bodily sensations (7), concerns about making lifestyle changes (7, 15), and difficulties associated with resuming work or being unable to work (7). Some survivors express difficulty in accepting the diagnosis and the disease itself (6). For some, the trauma associated with the cardiac event can trigger the resurfacing of past traumas or unresolved grief, with concomitant intrusive thoughts and nightmares (7). Fear about the future (8, 11, 13) and concerns about having to reprioritise goals and life plans in a foreshortened future (8, 11) have also been reported. Concerns about having another heart attack and fear of dying can also emerge (7, 10), as survivors are forced to confront their mortality, possibly for the first time in their life (7, 8).

These challenging emotions, changes and experiences that follow an acute cardiac event can all be conceptualized as “cardiac distress.” We have defined this multi-dimensional construct in previous publications:

*Cardiac distress is a persistent negative emotional state rather than a transient state; involving multiple psychosocial domains; that challenges a patient's capacity to cope with living with their heart condition, the treatment of the condition, and the resultant changes to daily living; and challenges the person's sense of self and future orientation* (16, 17).

Importantly cardiac distress spans multiple psychosocial domains, thereby incorporating patients' responses to physical, affective, cognitive, behavioral and social symptoms and experiences related to their cardiac event and their recovery (16). The definition also highlights both present and future concerns, thereby taking into account impacts on current self-perception as well as fears about the future (16).

Current understanding of the prevalence and correlates of cardiac distress has been limited by a lack of quantitative studies on this topic. While several qualitative studies have been undertaken to explore survivors' post-event experiences, as outlined earlier, no quantitative studies have investigated the broad range of cardiac distress experiences across multiple psychosocial domains, and incorporating both current and future-orientations.

### Aims of the Study

In the present study we assessed the prevalence and severity of a broad range of physical, affective, cognitive, behavioral and social symptoms of cardiac distress. We also investigated the correlates of distress by identifying the patient characteristics that predict these distressing experiences. This is the first study to assess the prevalence, severity and predictors of cardiac distress in this comprehensive way.

## Methods

### Item Generation and Distress Questionnaire Preparation

A broad pool of items was generated following a strict protocol which has been described in full elsewhere (17). In brief, the process involved three key steps. First, items included in instruments to measure cardiac anxiety, depression, quality of life and other cardiac-related constructs were reviewed, as were measures of distress used in the oncology and diabetes settings, with a view to creating a pool of cardiac distress-related items. Second, qualitative studies from the cardiac literature were reviewed to identify relevant constructs and generate further items. Third, the item pool was reviewed by an expert multidisciplinary committee of cardiac researchers and clinicians to identify missing constructs and fine-tune item wording. Following these steps, a set of 74 items addressing various issues and concerns was generated. These assessed issues and concerns across seven key conceptual domains, determined a priori by the project team, namely symptoms, self-perception, concerns about the future, negative affect, self-management, social functioning and role functioning. Participants reported on whether or not they had experienced each of 74 issues or concerns in the past 4 weeks by responding Yes or No for each item. For endorsed items, participants then reported on the level of distress associated with the issue, using a response scale where 0 = “no distress at all,” 1 = “slight distress,” 2 = “moderate distress,” and 3 = “severe distress.”

### Demographic, Medical, and Psychosocial Questions

Questions regarding demographic, medical and psychosocial characteristics were also included. Demographic information included age, sex, country of birth, education, and employment status. Medical information included event type, cardiac rehabilitation (CR) attendance (Y/N), cardiovascular risk factors (high blood pressure, high cholesterol, obesity, and positive family history), and significant comorbidity (diabetes, musculoskeletal conditions, chronic obstructive pulmonary disorder, cancer, stroke, and dementia). Psychosocial data included living alone (Y/N), partner status (partnered/unpartnered), presence of a close confidante (Y/N), loss of a close relative or friend in the past 12 months (Y/N; defined as “recent bereavement”), having been diagnosed with a mental health disorder prior to the cardiac event (Y/N, defined as “mental health history”), and financial strain reported on a 5-point Likert scale from 0 “no financial strain” to 4 “extreme financial strain.” The questionnaire was prepared in both hardcopy form for mailout and return, and *via* the REDCap (Research Electronic Data Capture) system for online completion.

### Inclusion and Exclusion Criteria

Eligible participants were those who have had an acute coronary event, namely acute myocardial infarction (AMI), percutaneous coronary intervention (PCI), coronary artery bypass graft surgery (CABGS), valve issues, heart rhythm disturbance, spontaneous coronary artery dissection (SCAD), or cardiac arrest in the previous 12 months and who attended an outpatient clinic at participating hospitals. Patients who did not have adequate English language proficiency to read and understand the PICF and questionnaire were excluded.

### Participant Recruitment

A sample of 194 patients was recruited from two hospitals in Australia, one in metropolitan Melbourne (Monash Health) and one in regional Victoria (Barwon Health, Geelong). The procedure at each of the two hospitals differed slightly.

At Barwon Health, the majority of participants were recruited while they were inpatients at the University Hospital Geelong following their hospital admission for either AMI, CABGS or PCI. At this contact, the Research Nurse provided eligible patients with a brief explanation of the study and asked if they would be willing to participate. Interested patients were then provided with the consent form. Consent was obtained to re-contact participants *via* telephone ~6–8 weeks later to complete the questionnaire. A smaller number of participants were recruited during their attendance at cardiac rehabilitation (CR).

At Monash Health, participants were recruited at the time of their appointment at the Cardiothoracic Preadmission Clinic, prior to their hospital admission for CABGS, or in the Cardiac Care Unit (CCU) for those with AMI, PCI, and other cardiac conditions. At this contact, the Research Nurse provided eligible patients with a brief explanation of the study and asked if they would be willing to participate. Interested patients were then provided with the consent form. Consent was obtained to re-contact participants at their routine 6–8-week follow-up appointment.

### Questionnaire Completion

Due to the COVID-19 pandemic and imposed lockdowns, participant recruitment varied between face-to-face and *via* telehealth. Questionnaires were either completed in hard copy or online. Thus, instead of completing the questionnaire while waiting for their clinic appointments as originally intended, participants were either directed to the website of the Australian Center for Heart Health (ACHH) to use an online link to the REDCap questionnaire or were mailed a hard copy of the questionnaire for completion at home and return in a reply-paid envelope to the ACHH. The questionnaire took ~25 min to complete. No identifying information was collected as no participant follow-up was involved.

### Ethics Approval

This study had ethics approval from the Monash Health Human Research Ethics Committee (approval number: RES-19-0000631A – 55979, which covered data collection at both the Monash Health and the Barwon University Hospital sites.

### Data Analysis

Frequencies were calculated for the prevalence and the distress severity ratings of each of the 74 issues and concerns. Items were clustered into the seven pre-determined domains of Symptoms (15 items), Self-perception (nine items), Concerns about the future (eight items), Negative Affect (eight items), Social functioning (16 items), Role functioning (five items), and Self-management (13 items). For prevalence, positively endorsed items within each domain were added together to provide domain prevalence scores. Within the “Symptoms” domain, two sub-domains of “physical symptoms” (seven items) and “cognitive symptoms” (five items) were also created. Higher domain and sub-domain prevalence scores indicated endorsement of more items (issues and concerns) within that domain. For distress severity ratings, a distress severity score for each domain was calculated by taking the mean severity ratings of endorsed items within that domain. For each domain and sub-domain, for both prevalence scores and distress severity scores, bivariate analyses (*t*-tests) were undertaken to identify variations in terms of demographic (age, sex, and employment status), medical (event type, CVD risk factors, comorbidities, and CR attendance), and psychosocial (mental health history, social isolation, and financial strain) characteristics. These particular psychosocial characteristics were selected for examination as they have been previously demonstrated to be strongly predictive of persistent or worsening post-event anxiety and depression (18). Using significant variables from the bivariate analyses, a series of multivariable linear regression analyses were then undertaken to identify the key predictors of prevalence and severity for each domain and sub-domain. For each model the variables entered included: age (years), sex, event type (CABGS vs. non-CABGS), living alone status, financial stress, history of mental illness, bereavement, and employment status. For the financial strain variable, responses of 3 “moderate,” 4 “considerable,” and 5 “extreme financial strain” were combined to indicate presence of financial strain. CVD risk factors (high blood pressure, high cholesterol, obesity, positive family history), various event types (AMI, PCI, and “other”), CR attendance, and comorbidities (Diabetes Mellitus, musculoskeletal, and “other” comorbid conditions) were not significant at the bivariate level and were therefore not entered into the multivariate analyses.

## Results

### Participants

Participants ranged in age from 22 to 90 years, with a mean (SD) age of 63.7 (11.2) years. Participant sociodemographic, psychosocial and medical characteristics are shown in Table 1.

**Table 1:** Characteristics of participants.

		***N***	***n***	**%**
**Sociodemographic characteristics**			
Sex	Male	194	140	72.2
	Female		53	27.3
	Prefer not to say		1	0.5
Country of birth	Australia	194	138	71.1
	Outside Australia		56	28.9
Marital status	Married/living with partner	194	137	70.6
	Divorced/separated		26	13.4
	Widowed		15	7.7
	Never married		16	8.2
Education	Primary	193	3	1.6
	Secondary		83	43.0
	Trade or certificate		55	28.5
	University degree/post-graduate		52	26.9
Employment status	In workforce	194	98	50.5
	Not in workforce		96	49.5
**Psychosocial characteristics**			
Lives alone		189	43	22.8
Recent bereavement		179	43	24.0
Financial strain		179	73	40.8
Mental health history		194	64	33.0
**Medical characteristics**			
Event type	Acute myocardial infarction	194	102	52.6
	Coronary artery bypass graft surgery		74	38.1
	Percutaneous coronary intervention		57	29.4
	Other		41	21.1
Attended cardiac rehabilitation		180	80	44.4
Significant co-morbidity		194	113	58.2

*N = 179–194 with variations due to incompletion of some questionnaires. Recent bereavement = having lost of relative or friend in the past 12 months. Financial strain = reports of moderate, considerable, or extreme financial strain. “Other” event type includes valve issues (n = 21), heart rhythm disturbance (n = 15), SCAD (n = 6), and cardiac arrest (n = 6)*.

As shown in Table 1, participants were mostly male (72%) and married or partnered (71%). Most (71%) were born in Australia and for those who were not (*n* = 56), the majority were born in the United Kingdom (*n* = 18), and New Zealand (*n* = 7). Nearly half of the participants were no longer in the paid workforce, and 40% were experiencing financial strain. Approximately one quarter lived alone and for those who lived with others (*n* = 146), the majority lived with their partner (*n* = 83), or their partner and children (*n* = 40).

All participants had experienced their event in the previous 12 months, with the majority (94%) occurring within the past 3 months (mean = 2.2, SD = 1.9 months). Most participants had an AMI, CABGS, or PCI, while a smaller number (21%) reported having experienced other heart issues (valve issues, *n* = 21; heart rhythm disturbance, *n* = 15; SCAD, *n* = 6; cardiac arrest, *n* = 6). More than half (*n* = 100, 56%) of the participants did not attend CR, with the most common reasons being people waiting for CR (42%), declining attendance (21%), and concerns associated with COVID-19 (14%), including barriers associated with using telehealth. Just over half the participants (58%) had at least one significant co-morbidity, most often diabetes (*n* = 58, 30%) or musculoskeletal conditions (*n* = 40, 32%). In addition, cardiovascular risk factors such as hypertension (37%), high cholesterol (31%), obesity (13%), and obstructive sleep apnoea (12%) were present. One third of participants had a history of mental health problems (depression or anxiety), with one in four having experienced depression and one in five having experienced anxiety.

### Most Common Issues and Concerns

**Figure 1:**
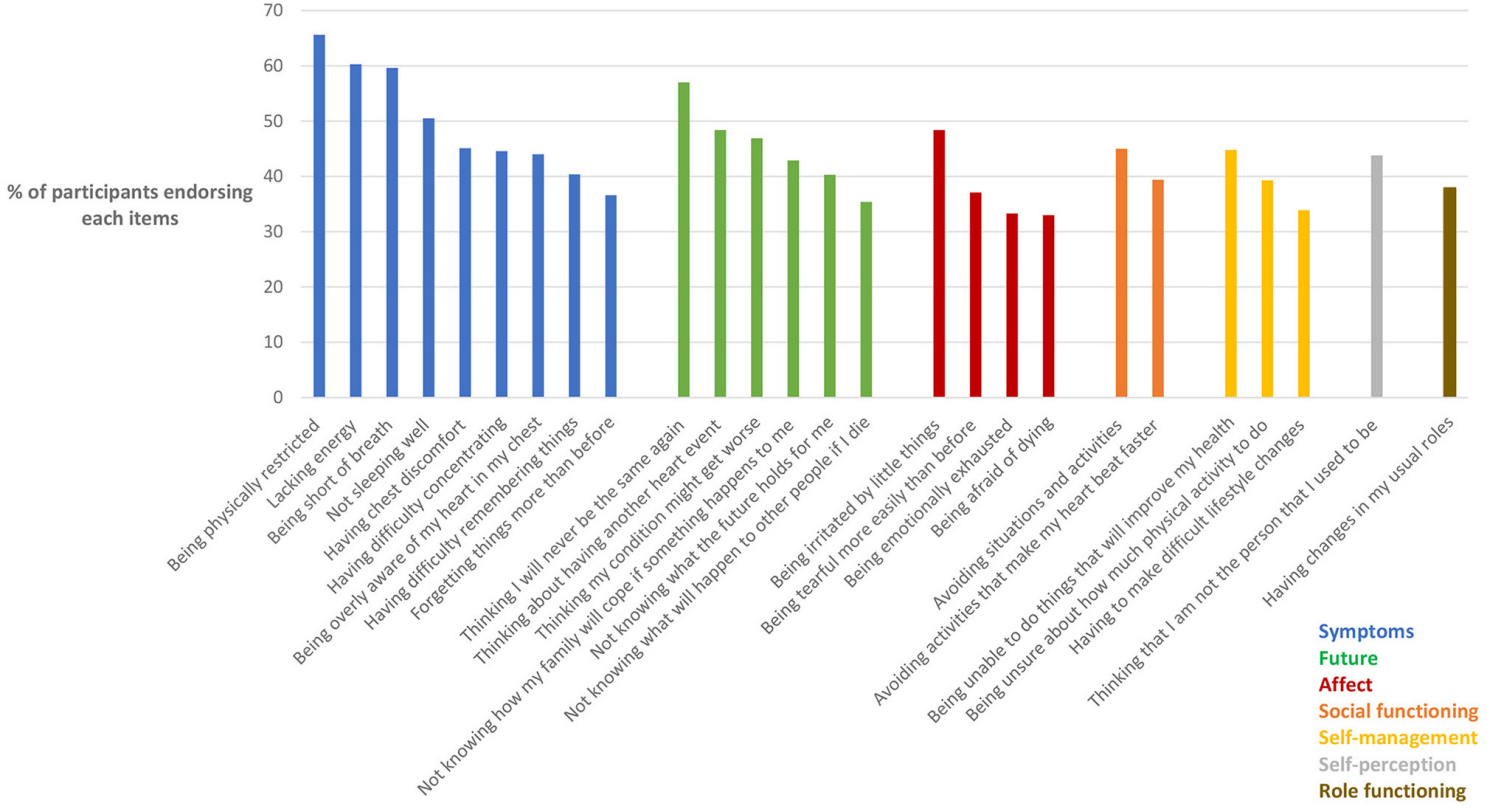
Prevalence of issues and concerns in domains. *N* = 189–194 with variations due to incompletions in some questionnaires. Only items endorsed by >33% of participants shown.

The most commonly endorsed issues and concerns are shown in Table 2 and Figure 1, with those items endorsed by over a third of participants depicted. The data indicate the proportion of participants who had experienced each of these in the previous 4 weeks. Table 2 shows the items in rank order from highest to lowest, while Figure 1 shows the same items grouped according to the seven pre-determined domains.

**Table 2:** Prevalence of issues and concerns in rank order.

**Issue/item**	***n***	**%**
Being physically restricted	126	65.6
Lacking energy	115	60.3
Being short of breath	115	59.6
Thinking I will never be the same again	110	57.0
Not sleeping well	98	50.5
Thinking about having another heart event	93	48.4
Being irritated by little things	93	48.4
Thinking my condition might get worse	91	46.9
Having chest discomfort	87	45.1
Avoiding situations and activities	86	45.0
Being unable to do things that I know will improve my health	87	44.8
Having difficulty concentrating	86	44.6
Being overly aware of my heart in my chest	85	44.0
Thinking that I am not the person that I used to be	84	43.8
Not knowing how my family will cope if something should happen to me	82	42.9
Having difficulty remembering things	78	40.4
Not knowing what the future holds for me	77	40.3
Avoiding activities that make my heart beat faster	76	39.4
Being unsure about how much exercise or physical activity I should be doing	75	39.3
Having changes in my usual roles	72	37.9
Being tearful more easily than before	72	37.1
Forgetting things more than before	70	36.6
Not knowing what will happen to other people if I die	68	35.4
Having to make difficult lifestyle changes because of my heart condition	65	33.9
Being emotionally exhausted	63	33.3
Being afraid of dying	64	33.0

*N = 189–194 with variations due to incompletions in some questionnaires*.

*Only items endorsed by >33% of participants shown*.

As shown in Table 2, the most commonly endorsed items were being physically restricted, lacking energy, being short of breath, thinking I will never be the same again, and not sleeping well. Each of these five items was endorsed by ≥50% of participants. A further 10 items were endorsed by between 40 and 50% of participants, including thinking about having another heart event, being irritated by little things, thinking my condition might get worse, having chest discomfort, avoiding situations and activities, being unable to do things that I know will improve my health, having difficulty concentrating, being overly aware of my heart in my chest, thinking that I'm not the person I used to be, and not knowing how my family will cope if something should happen to me. A further 11 items were endorsed by between 33 and 40% of participants. The remaining 48 items (from the full pool of 74 items) were each endorsed by fewer than 33% of participants (not shown in table).

As shown in Figure 1, four of the five most commonly endorsed items belonged to the “Symptoms” domain and more specifically, the “physical symptoms” sub-domain. Indeed, endorsement of items within the Symptoms domain was common, with nine of the symptom-related items each being endorsed by over a third of participants. Items within the “Future” domain were the next most commonly endorsed, with three items from this domain being in the top 10, and six being endorsed by over a third of participants. Items in the “Affect” domain were the next most prevalent, with four being endorsed by over a third of participants. In contrast, items within the domains of “Social functioning,” “Self-management,” “Self-perception,” and “Role functioning” were the least commonly endorsed.

### Most Distressing Issues and Concerns

While some items were highly prevalent, others were highly distressing. The items which elicited the highest ratings in terms of the severity of distress they caused are shown in Table 3. Not having access to needed health care was identified as the most distressing issue, being rated as either moderately or severely distressing by 74% of those who experienced this issue. Notably though, only 14% of participants actually reported experiencing this issue. Being concerned about capacity for sexual activity was the next most distressing issue, being rated as moderately or severely distressing by 64% of those who experienced it. However, only around one in five participants reported experiencing this issue. Being unsupported by family and friends, being isolated from family and friends, and being unavailable to family and friends were each reported as highly distressing albeit not commonly experienced issues. Notably, “not knowing how my family will cope if something happens to me” and “not sleeping well” were rated as both distressing and common.

**Table 3:** Most distressing issues and concerns in rank order.

	**Prevalence**	**Level of distress caused**		
		**Moderate**	**Severe**	**Moderate or severe**
Not having access to the health care I need	14.0	66.7	7.4	74.1
Being concerned about my capacity for sexual activity	22.0	47.6	16.7	64.3
Being unsupported by my friends or family	6.8	38.5	23.1	61.6
Being isolated from family and friends	15.3	44.8	13.8	58.6
Not knowing how my family will cope if something happens to me	42.9	39.0	18.3	57.3
Being unavailable to my family and friends	17.3	45.5	9.1	54.6
Not sleeping well	50.5	43.3	10.3	53.6
Having more pain than I expected to have	22.8	36.4	15.9	52.3
Having bad dreams and nightmares	24.0	23.9	28.3	52.2
Not being able to cope effectively with my heart condition	27.1	40.4	11.5	51.9
Lacking energy	60.3	36.0	15.8	51.8
Becoming a burden to my family	31.4	38.3	13.3	51.6
Not knowing what the future holds for me	40.3	42.9	7.8	50.7
Being unsure about how much exercise I should be doing	39.3	45.3	5.3	50.6
Being emotionally exhausted	33.3	42.9	6.3	49.2
Being unable to deal with stress	32.8	38.7	9.7	48.4
Being unable to do things that will improve my health	44.8	41.4	6.9	48.3
Being unable to plan for the future	24.9	39.6	8.3	47.9
Not being able to return to work or continue working	23.7	25.6	22.2	47.8
Thinking that I'm not the person I used to be	43.8	40.5	7.1	47.6
Not having anyone to talk to who understands my difficulties	17.9	35.3	11.8	47.1
Being afraid of dying	33.0	35.9	10.9	46.8
Feeling lonely	24.5	38.3	8.5	46.8
Lacking purpose and meaning in life	27.1	28.8	17.3	46.1
Having difficulty making decisions	25.0	4.2	4.2	45.9

*N = 189–194 with variations due to incompletions in some questionnaires. Only 25 most distressing items shown*.

In terms of the experience of “severe distress,” having bad dreams and nightmares was identified as the most severely distressing for those who experienced it; 28% of those who experienced bad dreams and nightmares reported that this was “severely distressing.” Being unsupported by family and friends and being unable to work were also commonly identified as causing severe distress amongst those who experienced these issues.

**Figure 2:**
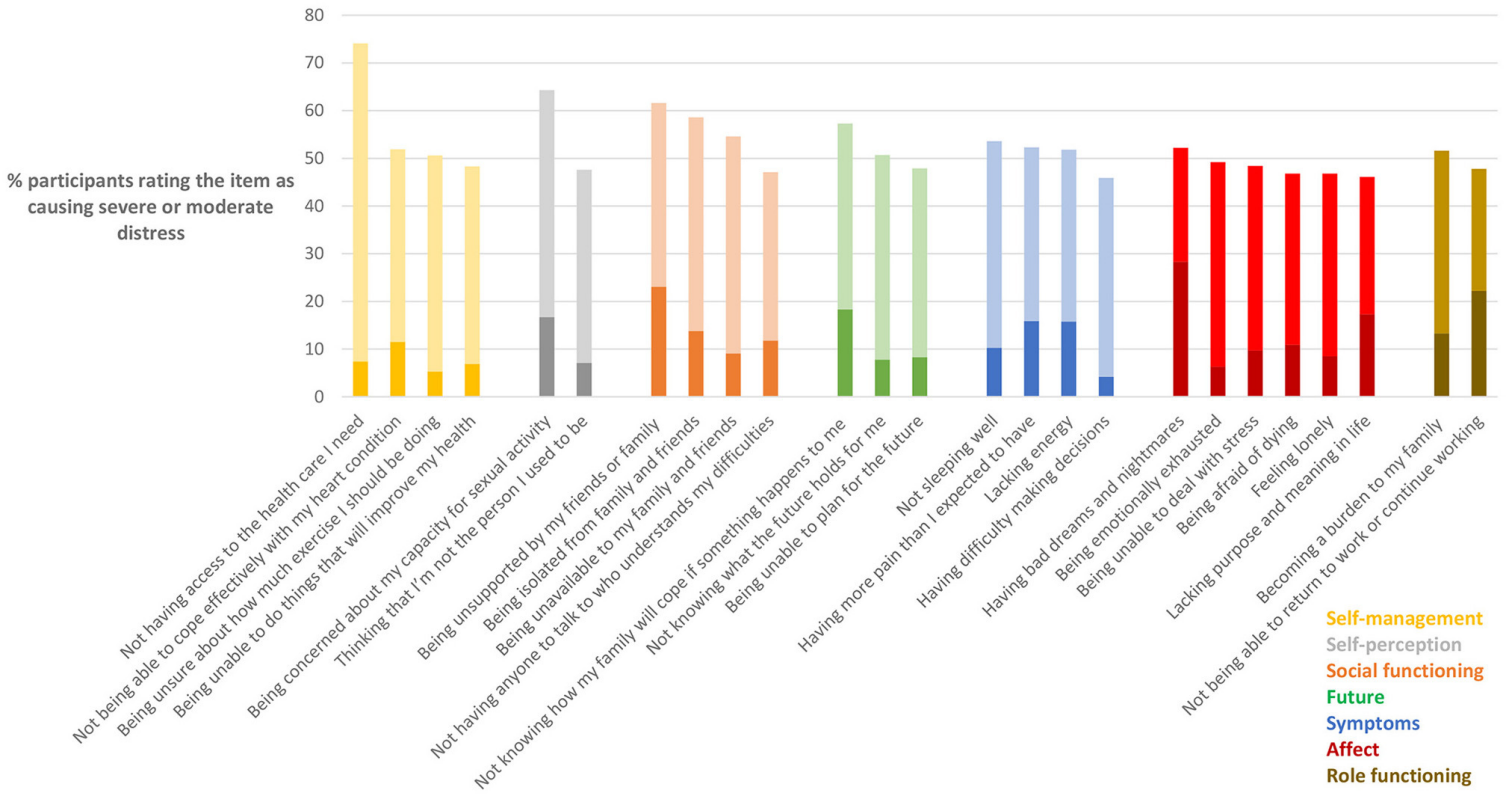
Issues and concerns rated as severely or moderately distressing. *N* = 189–194 with variations due to incompletions in some questionnaires. Dark color indicates rating of “severe” distress, light color indicates rating of “moderate distress.” Only 25 most distressing items shown.

As shown in Figure 2, the two most distressing issues belonged to the “Self-management” and “Self-perception” domains, although other items within these two domains were rated as less distressing. Three of the four next most distressing items belonged to the “Social” domain. For both the “Social” and “Symptoms” domains, three items were rated as moderately or severely distressing by over 50% of those who experienced these issues, whereas this was the case for only one item within each of the “Future,” “Affect,” and “Role functioning” domains. Overall though, the pattern evidenced in Figure 2 shows that a range of items across all six domains were identified as highly distressing.

### Predictors of Prevalence and Severity Ratings

Results of the multivariate regression analyses are shown in Table 4.

**Table 4:** Significant predictors of distress prevalence and severity ratings.

		**Predictor variables**							
	**Female sex**		**CABGS**		**Live alone**		**Financial Strain**		**History of mental illness**	
	**aOR (95%CI)**	***p***	**aOR (95%CI)**	***p***	**aOR (95%CI)**	***p***	**aOR (95%CI)**	***p***	**aOR (95%CI)**	***p***
**Prevalence ratings**										
Symptoms	4.29 (1.33–13.87)	0.015	2.97 (1.33–13.87)	0.042			6.95 (2.46–19.67)	<0.001	5.81 (1.95–17.33)	0.002
Sympt-Phys	1.89 (1.02–3.49)	0.042	1.98 (1.14–3.43)	0.015			2.27 (1.31–3.93)	0.004	2.15 (1.21–3.81)	0.009
Sympt-Cog					1.92 (1.08–3.44)	0.027	1.90 (1.17–3.07)	0.009	2.01 (1.21–3.34)	0.007
Future	2.45 (1.01–5.97)	0.049					3.73 (1.69–8.22)	0.001		
Affect	2.17 (1.06–4.47)	0.035					3.64 (1.91–6.90)	<0.001	5.12 (2.60–10.08)	<0.001
Social	3.82 (1.21–12.07)	0.023			6.29 (1.86–21.29)	0.003	6.66 (2.42–18.30)	<0.001	5.58 (1.92–16.12)	0.002
Self-perception							3.84 (1.92–7.71)	<0.001	2.47 (1.17–5.22)	0.018
Role function							2.44 (1.57–3.80)	<0.001		
Self-manage							8.38 (3.33–21.10)	<0.001	3.11 (1.17–8.30)	0.024
**Severity ratings**										
Symptoms									1.42 (1.18–1.72)	<0.001
Sympt-Phys									1.42 (1.15–1.76)	0.002
Sympt-Cog									1.45 (1.11–1.89)	0.007
Future									1.35 (1.08–1.69)	0.009
Affect							1.25 (1.02–1.54)	0.032	1.40 (1.13–1.74)	0.002
Social									1.53 (1.25–1.96)	<0.001
Self-perception									1.45 (1.12–1.87)	<0.001
Role function									1.59 (1.15–2.19)	0.005
Self-manage							1.29 (1.06–1.57)	0.012		

*Full multivariable linear regression models for each rating included: age (years), sex, CABGS status, live alone status, financial stress, history of mental illness, and bereavement in past 12 months. aOR, adjusted odds ratio; CABGS, coronary artery bypass graft surgery; Symptoms, symptoms domain; Sympt-Phys, Physical symptoms subdomain; Sympt-Cog, Cognitive symptoms subdomain; Future, Concerns about the future domain; Affect, Negative Affect domain; Social, Social functioning domain; Self-Perception, Self-perception domain; Role Function, Role functioning domain; Self-Manage, Self-management domain*.

In terms of prevalence ratings, financial strain was a significant predictor for all domains, and history of mental illness was a significant predictor for all but two domains. Living alone was predictive of prevalence ratings for the “Cognitive symptoms” sub-domain and the “Social” domain. Females had significantly higher prevalence for the “Symptoms,” “Future,” “Affect,” and “Social” domains, and the “Physical symptoms” sub-domain, while those who had undergone CABGS had higher prevalence for the “Symptoms” domain and the “Physical symptoms” sub-domain. Bereavement was associated with higher prevalence for the Symptoms domain [aOR = 3.82 (95% CI 1.19–12.33), *p* = 0.025], while employment status was not significantly associated with prevalence ratings for any of the domains (not shown in table).

In terms of severity ratings, history of mental illness was a significant predictor across all but one domain, while financial strain was a significant predictor for the “Affect” and “Self-management” domains. Younger age was associated with higher severity ratings for the “Social” domain [aOR = 0.99 (95% CI 0.97–1.00), *p* = 0.016; not shown in table]. Female sex, event type, living alone, and employment status were not significantly associated with severity ratings for any of the domains.

## Discussion

The findings of the present study document the prevalence and severity of cardiac distress across 74 items within seven domains relating to cardiac symptoms, self-perception, concerns about the future, negative affect, social functioning, role functioning, and self-management. The most commonly endorsed items were within the domains of dealing with symptoms, fear of the future, negative affect, and social functioning, and included “being physically restricted,” “lacking energy,” “being short of breath,” “thinking I will never be the same again,” and “not sleeping well.” While less prevalent, “not having access to the health care I need,” “being concerned about my capacity for sexual activity,” and “being unsupported by family and friends” were reported as highly distressing for those experiencing these issues. The study has also identified key demographic and psychosocial predictors of distress. In doing so, the study has expanded our understanding of the multi-faceted nature of distress caused by a cardiac event.

Many of the highly prevalent issues and concerns identified by these cardiac event survivors related to physical symptoms and the fear that they would not ever be the same again. Concerns about loss of health have been identified previously in qualitative studies (6, 8, 11), as have concerns about loss of one's previous sense of self (13). Many of these concerns about symptoms and physical adjustment are addressed and normalized in CR, thereby reinforcing the need to increase the rate of referral to and attendance at CR as a crucial step in recovery.

Concerns about the future were also highly prevalent. These included fears about worsening symptoms, having a recurrent event and, ultimately, fears about death. Again, these issues have been identified in previous qualitative studies (7, 8, 10, 11, 13). Additionally, future-related items were also identified as highly distressing, particularly those centering on the issue of an uncertain future. Previous qualitative studies have similarly highlighted the distress caused by uncertainty and the inherent inability to plan for the future post-cardiac event (8–11).

While recognizing the highly prevalent nature of physical symptoms in particular, this study highlights the importance of assessing not just what people experience, but how distressing these issues are perceived to be. Not having access to needed health care was identified as the most distressing issue, extending previous qualitative findings regarding worries about obtaining professional help and advice (9, 13). Given that the present study was conducted during the COVID-19 pandemic, with concomitant restrictions in face-to-face healthcare delivery and pivoting to telehealth support options, it is perhaps not surprising that this issue was identified as highly distressing. Also extending previous qualitative studies (7, 14), ours is the first to highlight concerns about sexual capacity as amongst the most distressing of all issues faced by cardiac event survivors. Concern about future sexual capacity reflects not only a person's concern with physical functioning but represents an important dimension of personal identity that is under threat, and a concern with acceptance of potential limitations, whether psychological or physical in origin.

The present findings highlight a number of issues that can be addressed in cardiac rehabilitation. Difficulties due to sleep disturbance, including poor sleep quality and nightmares, and concerns about how patients' families will cope, were both highly endorsed and very distressing. These issues are very important to address, to enhance patients' physical and emotional recovery. We have argued elsewhere for the need for screening for sleep disorders in the recovery phase during CR (19) so that survivors are given relevant and timely assistance with this issue. Similarly, health professionals engaging with cardiac patients in recovery need to be alert to survivors' concerns about family and, indeed, to the importance of knowing about the degree of connectedness survivors have with their family or within their community. Those experiencing difficulties in this area should be considered for referral to a family-oriented service.

It is notable that women experienced more of the issues and concerns listed than did men, with female sex a significant predictor of high prevalence across several domains. This is consistent with women's previously reported higher levels of anxiety and depression (20) and may also be due to women being more expressive than men and highly attuned to identifying and acknowledging their psychosocial concerns. Although female sex was not predictive of distress severity ratings, this may be due to the fact that these analyses were undertaken by domain rather than for individual items. Consistently, it was somewhat surprising that few age differences were identified and, again, this may be due to a lack of fine-tuned comparisons, which was outside the scope of the present study.

As may be expected, people with a mental health history experienced more of the issues and concerns, and also rated them as more distressing. Those with a mental health history tend to have a lower base of resilience and coping and therefore will experience more cardiac-related issues and find them inherently more distressing. Previous anxiety/depression is a known risk factor for poor post-cardiac event mental health outcomes (3, 18), underscoring the importance of identifying these patients early on and targeting them for mental health support.

The present study also identified other known correlates of persistent anxiety and depression such as financial strain (3, 18), and social isolation (3, 18). Indeed, financial strain was a consistent predictor of prevalence ratings across all domains and sub-domains, highlighting this patient characteristic as an important and easily assessed “red flag” for poor post-event mental health recovery, as we have noted previously (3, 18). Feelings of isolation have been exacerbated by the COVID–related mandatory lockdowns and restrictions, and have brought this issue into sharp focus internationally (21). Poignantly, of those who reporting feeling unsupported by family and friends, over 60% found this severely or moderately distressing, with this distress likely heightened by the COVID-19 lockdowns (21). Social isolation as a barrier to good recovery has been identified as a contributor to persistent or worsening mental health post cardiac event (3, 22) as well as conferring a higher risk of premature death (23). Those who had experienced a recent bereavement endorsed more items related to cognitive symptoms, raising concerns for this group in terms of their cardiac recovery. Consistently, a recent study found that death of a spouse or partner during the year prior to a first AMI is associated with an increased risk of recurrent AMI and cardiac-related death (24), highlighting bereavement as a red flag for compromised cardiac recovery.

### Limitations

Some study limitations should be acknowledged. First, while the present results confirm our definition of cardiac distress as multifactorial and non-transient, a stronger case for the latter point may have been made if the questionnaire had been administered further into convalescence. The 8-week assessment point was chosen to coincide with routine follow-up clinic appointments, in order to optimize participation. However, at this relatively early point of recovery it is possible that symptoms of the cardiac blues, typical during the early post-event adjustment period (25), may not yet have resolved. Future longitudinal studies to investigate the trajectories of the components of distress are needed. Second, due to the large number of distress items assessed, we identified predictors for domains only, rather than for individual items. More fine-tuned analysis to investigate item correlates would provide a more nuanced understanding of sex and age-related differences in particular, but was outside the scope of the present study. Again, this could be the focus of more targeted hypothesis testing within specific distress domains and/or for specific patient groups. Third, being undertaken during the COVID-19 pandemic, the study findings may have been impacted by transient and extraneous stressors that were not measured. Despite these limitations, the study was strengthened by the inclusion of consecutive series of cardiac patients from two large hospitals, representing both metropolitan and regional areas of Australia.

## Conclusion And Clinical Implications

The present study extends the findings of earlier qualitative studies by quantifying common issues and concerns experienced by cardiac event survivors and providing assessment of the level of distress caused by these issues and concerns. It is the first study to explore cardiac distress in this way. The findings highlight the importance of investigating both endorsement of these issues as well as ratings of distress severity in our attempts to understand cardiac distress. The fact that issues such as not being able to access suitable health care and concern about sexual functioning were highly distressing although not highly endorsed, shows the necessity for measuring both, and not simply assuming that high prevalence of an issue corelates with a high level of distress and vice versa. In terms of clinical implications, the findings underscore the importance of providing the opportunity for patients to express the specific nature of their worries and concerns, and to reveal the impacts of these on their psychological wellbeing. Patients expressing high levels of distress should be given the opportunity for psychocardiology-informed counseling to support them in their recovery. Indeed, the findings point to the need for implementing routine screening for cardiac distress in primary care and cardiac rehabilitation settings. The present findings further our understanding of the relative importance of various post-event issues and concerns, thereby providing useful clinical guidance for health professionals working in cardiac rehabilitation and psychocardiology settings.

## Data Availability Statement

The raw data supporting the conclusions of this article will be made available by the authors, without undue reservation.

## Ethics Statement

This study had ethics approval from the Monash Health Human Research Ethics Committee (approval number: RES-19-0000631A – 55979). Written informed consent for participation was not required for this study in accordance with the national legislation and the institutional requirements.

## Author Contributions

BM, MR, ML, and AJ undertook data analysis and data interpretation. AJ, MR, and BM drafted the manuscript. JA, JS, VH, MA, RH, CS, and DT contributed to and approved the manuscript. All authors contributed to study design, questionnaire development, and data collection. All authors contributed to the article and approved the submitted version.

## Funding

This study was partially funded by the Angela Anita Reid bequest to the Australian Centre for Heart Health.

## Conflict of Interest

The authors declare that the research was conducted in the absence of any commercial or financial relationships that could be construed as a potential conflict of interest.

## Publisher's Note

All claims expressed in this article are solely those of the authors and do not necessarily represent those of their affiliated organizations, or those of the publisher, the editors and the reviewers. Any product that may be evaluated in this article, or claim that may be made by its manufacturer, is not guaranteed or endorsed by the publisher.
